# Effects of Chrysin and Chrysin-7-sulfate on Ochratoxin A-Albumin Interactions and on the Plasma and Kidney Levels of the Mycotoxin in Rats

**DOI:** 10.1021/acsomega.4c01738

**Published:** 2024-04-02

**Authors:** Miklós Poór, Ágnes Dombi, Eszter Fliszár-Nyúl, Lorenzo Pedroni, Luca Dellafiora

**Affiliations:** † Department of Laboratory Medicine, Medical School, University of Pécs, Ifjúság útja 13, Pécs H-7624, Hungary; ‡ Molecular Medicine Research Group, János Szentágothai Research Centre, University of Pécs, Ifjúság útja 20, Pécs H-7624, Hungary; § Department of Pharmacology, Faculty of Pharmacy, University of Pécs, Rókus u. 2, Pécs H-7624, Hungary; ∥ Department of Food and Drug, University of Parma, Via G.P. Usberti 27/A, Parma 43124, Italy

## Abstract

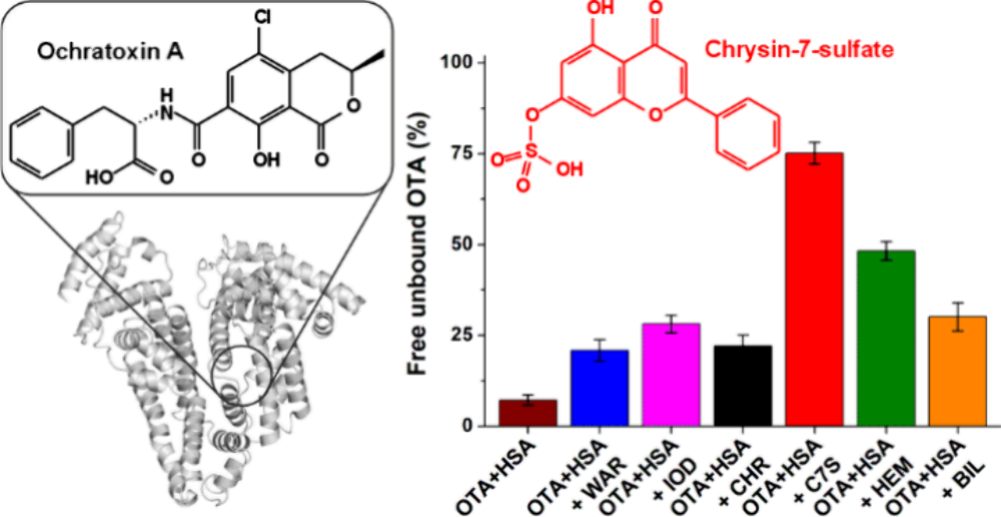

The nephrotoxic mycotoxin ochratoxin A (OTA) is a common food contaminant. OTA binds to the Sudlow’s Site I region of serum albumin with very high affinity, resulting in its slow elimination. The displacement of OTA from albumin may be beneficial due to the faster excretion of the mycotoxin, while it may also lead to the increased tissue uptake of OTA. Furthermore, it is challenging to displace the mycotoxin from albumin even with high-affinity Site I ligands. In this study, we tested the impacts of Site I and Heme site ligands on OTA-albumin interactions by applying fluorescence spectroscopic, ultracentrifugation, and modeling studies. Chrysin-7-sulfate (C7S) strongly displaced OTA from both human and rat albumins; therefore, the impacts of C7S (single intravenous administration) and the parent flavonoid chrysin (repeated peroral treatment) were examined on the plasma and kidney levels of OTA in rats. Chrysin barely influenced the concentrations of mycotoxin in plasma and kidneys. In the first few hours, C7S significantly decreased the plasma levels of OTA compared to the control animals; while after 24 h, only minor differences were noticed. Our study highlights the superior displacing ability of C7S vs OTA regarding human and rat albumins.

## 1 Introduction

**Figure 1:**
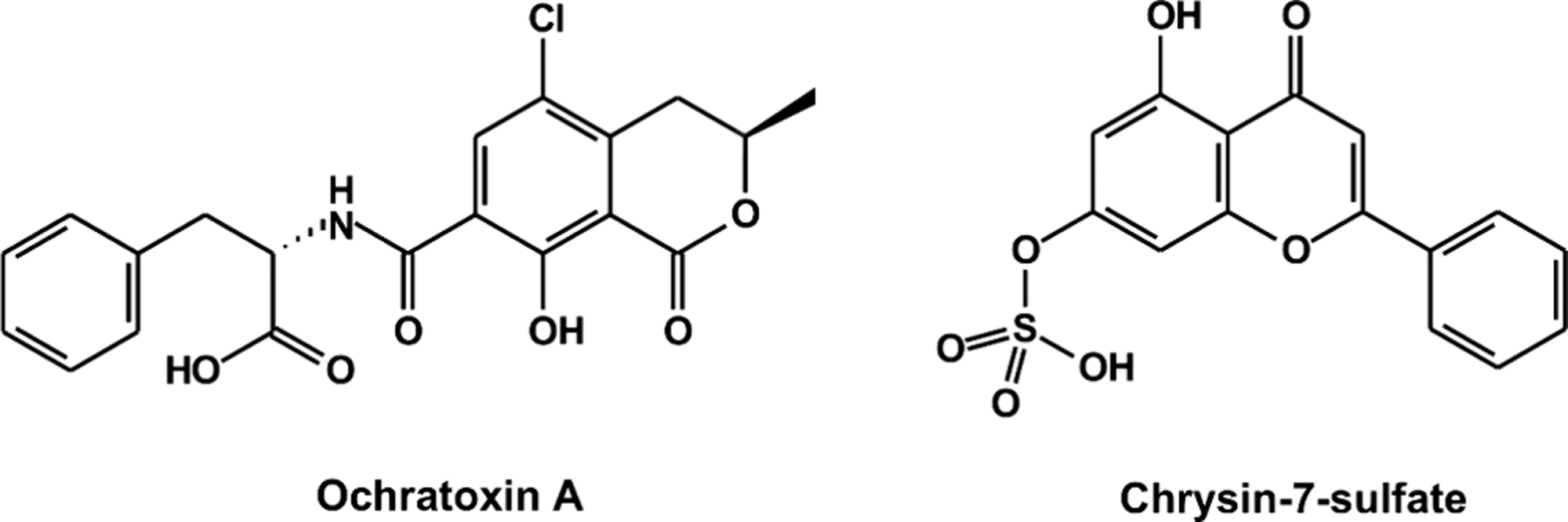
Chemical structures of mycotoxin ochratoxin A (OTA) and the flavonoid metabolite chrysin-7-sulfate (C7S).

Ochratoxin A (OTA; Figure 1) is a mycotoxin produced by *Aspergillus* and *Penicillium* filamentous fungi.^1^ OTA is a common contaminant in several foodstuffs, including cereals, meat and dairy products, fruits, oilseeds, coffee beans, and beverages (e.g., coffee, tea, milk, wine, and beer).^1−3^ The major target organs of OTA are the kidneys leading to its nephrotoxic impact; however, the high chronic exposure to the mycotoxin may also result in hepatotoxic, neurotoxic, immunotoxic, teratogenic, and carcinogenic effects.^1,3^ The International Agency for Research on Cancer (IARC) classifies OTA as “possibly carcinogenic to humans” (Group 2B).^4^ OTA has high oral bioavailability and long elimination half-life in humans; the latter is primarily resulted from the strong albumin binding and the low rate of metabolism of the mycotoxin.^3^

Serum albumin is the main carrier of several endogenous compounds, drugs, and toxins in the circulation, affecting their pharmacokinetic/toxicokinetic properties.^5,6^ There are three major drug binding sites on albumin: Sudlow’s Site I (or FA7, in subdomain IIA), Sudlow’s Site II (or FA3-FA4, in subdomain IIIA), and Heme site (also called as Heme pocket or FA1, in subdomain IB).^5−7^ The typical ligands of Site I are for example warfarin, iodipamide, phenylbutazone, and furosemide.^6^ Diazepam, ibuprofen, and naproxen are well-known ligands of Site II.^6^ Furthermore, bilirubin, biliverdin, and hemin bind to the Heme site with high affinity.^5,7^

Both experimental (displacement experiments with site markers, interaction with recombinant albumin fragments, and site-directed mutagenesis) and modeling studies demonstrated that the high-affinity binding site of OTA is located in Site I (subdomain IIA).^8,9^ Human serum albumin (HSA) contains only one tryptophan amino acid (W214) in Site I.^5^ Fluorescence spectroscopic experiments demonstrated the energy transfer between W214 and OTA, also representing that the binding site of the mycotoxin is close to this tryptophan moiety.^10^ As previous studies highlighted, dianionic OTA (both carboxyl and phenolic hydroxyl groups of the mycotoxin are deprotonated) forms highly stable complexes with HSA: besides the apolar–apolar interactions with the hydrophobic Site I cavity, OTA-HSA is further stabilized by ionic interactions with R257 and R218 arginines.^9,11^ The association/binding constant of the OTA-HSA complex exceeds 10^7^ L/mol.^10,12^ The formation of highly stable OTA-HSA complexes results in the very low free unbound fraction of the mycotoxin in the human circulation (0.02%) and its long plasma elimination half-life in man (approximately 35 days).^3^ Due to the strong interaction of OTA with HSA, it is challenging to displace the mycotoxin from albumin even with the high-affinity ligands of Site I (e.g., warfarin, furosemide, or phenylbutazone).^13^ Furthermore, OTA-albumin interaction shows high species-dependent differences.^14,15^ Bovine serum albumin (BSA) or rat serum albumin (RSA) form approximately 10-fold weaker complexes with the mycotoxin compared to HSA; nevertheless, the binding constants of OTA-BSA and OTA-RSA complexes are also very high (>10^6^ L/mol).^12^ The importance of albumin in the toxicokinetics of OTA has also been emphasized by the observation that the excretion of the mycotoxin is 20- to 70-fold faster in albumin-deficient than in normal rats.^16^ A recent study also examined the impacts of hypoalbuminemia on the tissue distribution of OTA, where the intravital imaging technique was applied to test the uptake of the mycotoxin into the livers and kidneys of albumin knockout and wild-type mice.^17^ Besides the faster biliary and urinary excretion of mycotoxin, the increased uptake of OTA into the liver and kidneys was noticed in albumin knockout mice. Nevertheless, we need to consider the limitations of the latter study, where a high dose of OTA (5 mg/kg) was administered intravenously,^17^ which may strongly enhance the nonspecific tissue uptake of the mycotoxin. Based on earlier studies, organic anion transporters (OATs)^18,19^ and organic anion transporting polypeptides (OATPs)^20,21^ are involved in the active uptake of OTA into kidney and liver cells, respectively. However, the high levels of OTA may saturate these uptake mechanisms, and the passive transport can become significant. In addition, considering the very high affinity of OTA toward albumins, it is unlikely that we can produce similarly strong *in vivo* impact with the displacement of the mycotoxin than the remarkable changes produced in albumin knockout animals. Since the disruption of OTA-albumin interaction can cause faster excretion and/or increased tissue uptake of the mycotoxin, the question remains: What is the major outcome of the partial displacement of OTA from serum albumin?

Chrysin (CHR) is a flavonoid aglycone; it is abundant in nature and contained at high doses in certain dietary supplements.^22,23^ Due to the high presystemic biotransformation of CHR in enterocytes and hepatocytes, its conjugated metabolites chrysin-7-sulfate (C7S; Figure 1) and chrysin-7-glucuronide (C7G) reach high concentrations in the circulation.^24,25^ In our earlier study, we demonstrated that C7S can strongly displace the Site I marker warfarin form HSA using fluorescence spectroscopic and ultrafiltration methods.^22^ In addition, fluorescence anisotropy studies suggested that C7S can also disrupt the albumin binding of OTA, while CHR and C7G caused only minor effects.^22^ Another fluorescence anisotropy-based experiment indicated that the Heme site marker bilirubin may be able to strongly decrease the albumin-bound fraction of OTA.^13^ Since Heme pocket and Site I are allosterically coupled,^5^ Heme site ligands can modulate the binding affinity of certain Site I ligands,^26^ which explains the potential impact of bilirubin on OTA-HSA interaction. Nevertheless, in our recent study, ultrafiltration and ultracentrifugation measurements demonstrated that fluorescence spectroscopic studies can show misleading results when two ligands are cooperatively bound to the protein at different binding sites.^26^ Therefore, confirmatory measurements with other techniques seem to be highly reasonable.

Considering the high importance of albumin in the toxicokinetics of OTA, we were curious about how we can modulate the albumin binding of the mycotoxin under *in vitro* and *in vivo* conditions. Therefore, in the current study, the impacts of Site I (warfarin, iodipamide, CHR, and C7S) and Heme site (hemin and bilirubin) ligands were tested on OTA-HSA and OTA-RSA interactions employing steady-state fluorescence spectroscopy, fluorescence anisotropy, and ultracentrifugation experiments. Furthermore, the influence of CHR and C7S on plasma and kidney levels of OTA were also examined in animal experiments (Wistar rats). Finally, molecular modeling studies were performed for a deeper understanding of the displacing effects of C7S and CHR regarding HSA and RSA. Our results demonstrate that C7S can effectively displace OTA from albumin both *in vitro* and *in vivo*, but it only slightly affects the toxicokinetics of the mycotoxin likely due to compensatory mechanisms.

## 2 Materials and Methods

### 2.1 Reagents

Ochratoxin A (OTA), chrysin (CHR), warfarin, iodipamide, hemin, bilirubin, human serum albumin (HSA; product code: A1653), and rat serum albumin (RSA; product code: A6272) were purchased from Merck (Darmstadt, Germany). Chyrsin-7-sulfate (C7S) was synthesized as it has been previously reported.^22,27^ All other reagents were analytical grade or HPLC grade.

### 2.2 Spectroscopic Studies

Fluorescence spectroscopic studies were carried out using a Hitachi F-4500 spectrofluorometer (Tokyo, Japan). Fluorescence emission spectra were collected, and fluorescence anisotropy values were determined in phosphate-buffered saline (PBS, pH 7.4) at room temperature, using 393 and 446 nm as excitation and emission wavelengths, respectively. To test the impacts of Site I and Heme site ligands on the OTA-albumin interaction, increasing amounts of warfarin, iodipamide, CHR, C7S, hemin, or bilirubin (0–30 μM) were added to OTA (1.0 μM) and albumin (1.5 μM). Absorption spectra of ligand molecules were also recorded employing a Jasco V730 UV–vis spectrophotometer (Tokyo, Japan), after which their inner-filter effects were corrected using the following equation:^28,29^
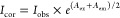
1where *I*_cor_ and *I*_obs_ are the corrected and the observed fluorescence emission intensities at 446 nm, respectively, while *A*_ex_ and *A*_em_ are the absorbance values of the competitor ligand molecules at 393 and 446 nm, respectively.

Fluorescence anisotropy (*r*) values were calculated employing the following equation:^10,30^
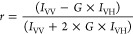
2where *I*_VV_ is the emission intensity measured in vertical positions of the polarizers at both the presample and the postsample sites, *I*_VH_ is the emission intensity measured in vertical position of the polarizer at presample site and in horizontal position of the polarizer at the postsample site, while *G* is the instrumental factor (showing the preference of the emission optics for the horizontal orientation to the vertical orientation).

### 2.3 Ultracentrifugation Studies

Albumin (with the bound ligand molecules) was sedimented with ultracentrifugation, employing the previously described method.^26,29,31^ Briefly, samples contained OTA (1.0 μM) and albumin (1.5 μM) without or with competitors (warfarin, iodipamide, CHR, C7S, hemin, or bilirubin) in PBS (pH 7.4). These solutions (900 μL) were centrifuged for 16 h at 170,000*g* and 20 °C, using an Optima MAX-XP tabletop ultracentrifuge (with an MLA-130 fixed-angle rotor; Beckman Coulter, Brea, CA, US). After 200 μL of the protein-free supernatant was carefully aspirated, the free unbound concentration of OTA was directly analyzed from the supernatant by HPLC-FLD (details in Section 2.6). Association constants (*K*_*a*_) of OTA-HSA and OTA-RSA complexes were calculated based on the following equation, assuming 1:1 stoichiometry of the complex formation:^29,32^
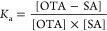
3where [OTA] is the concentration of the unbound free mycotoxin, [SA] is the concentration of the unbound free serum albumin, while [OTA-SA] is the molar concentration of the mycotoxin-albumin complex in the solution.

### 2.4 Modeling Studies

#### 2.4.1 Protein Model Preparation

The 3D model of HSA was obtained from the chain A of the crystallographic structure deposited on the Protein Data Bank (PDB; https://www.rcsb.org/)^33^ with PDB ID 1AO6.^34^ The residues were thoroughly checked, and the “Wizard → Mutagenesis” tool available on PyMol (v. 2.5.0) was used to fill side chain atoms when missing.

The 3D model of RSA was obtained through AlphaFold (https://alphafold.ebi.ac.uk/)^35^ browsing for the UniProt code P02770 (https://www.uniprot.org/).^36^ This was done since no structures were available on the PDB at the time of the analysis (last database access, November 20, 2023).

#### 2.4.2 Molecular Docking Simulation

Molecular docking simulations were performed through GOLD (Genetic Optimization for Ligand Docking; version 2022)^37^ using the internal GOLDScore scoring function (the higher the score, the more likely is the predicted binding pose), as it proved a high reliability to study serum albumin–ligand interactions.^38^ The docking protocol was set based on previous studies^39^ with semiflexible protein with polar hydrogens able to rotate freely and fully flexible ligands. There were two binding sites, both defined as a 5 Å radius sphere around two distinct centroids. The first centroid was set according to Rimac and co-workers^40^ and crystallographic data^41^ in a region of the Sudlow’s Site I flanking that occupied by OTA. CHR, C7S, and C7G were docked within this binding site in both HSA and RSA. The second centroid was set at Site I with OTA being docked therein, with either ligand-free HSA and RSA, or with HSA and RSA complexed with alternatively CHR, C7S, or C7G.

The thermodynamic stability of the generated complexes was estimated with HADDOCK Prodigy Web server,^42^ in agreement with our previous study.^43^

#### 2.4.3 Molecular Dynamics Simulation

Conventional molecular dynamics (CMD) simulations and steered dynamics simulations (SMD) were run on HSA complexes through GROMACS (v. 2019.4).^44^ For both procedures, the system was parametrized with a CHARMM27 all-atom force field^45^ and the ligand parametrization was performed on the CGenFF Web server (https://cgenff.silcsbio.com/).^46^ The system was solvated with SPCE waters in a cubic periodic boundary condition and neutralized adding Na^+^ and Cl^–^ as counterions. Prior to the production phase, an energetic minimization of the system was performed to eliminate steric clashes and rectify improper geometries. To do so, a steepest descent algorithm with a maximum of 5000 optimization steps was used. This was followed by an isothermal simulation at 300 K, and an isobaric simulation at 1 bar both with a coupling time of 2 ps and lasting for 100 ps, allowing the system to reach an equilibrium state.

Regarding CMD, the production phase lasted for 40 ns. On the other hand, SMD lasted 500 ps allowing to see the outward pathway and the rupture force for OTA differently complexed with CHR, C7S, and C7G. Specifically, SMD simulations were run through CHAPERONg^47^ setting two pull groups (OTA as mobile group and HSA as immobile group) along the *z*-axis. The pulling occurred at a pace of 0.01 nm/ps with a 4000 kJ × mol^–1^ × nm^–2^ pulling force.

### 2.5 Animal Studies

#### 2.5.1 Animals and Treatments

Male Wistar rats were obtained from Toxi-Coop Ltd. (Budapest, Hungary). The animals (weighing 150–250 g) were kept at 24 ± 2 °C, at 50–60% relative air humidity, and on a 12 h light/dark cycle in the Laboratory Animal House of the Department of Pharmacology and Pharmacotherapy (Medical School, University of Pécs) under standard pathogen-free conditions and were provided with standard rat chow and tap water *ad libitum*.

Stock solution of OTA (10 mg/mL) was prepared in 96 v/v% ethanol (Reanal, Budapest, Hungary). C7S (90 mg/mL) were dissolved in dimethyl sulfoxide (Fluka, Charlotte, NC, US). In the *i.v.* experiments, the final concentration of dimethyl sulfoxide was uniformly 1.67 v/v % in each solution administered. In the *per os* experiments, CHR was suspended in physiological saline by using a mortar and pestle.

In the first experiment, rats were weighed and then treated intravenously through the tail vein with OTA (50 μg/kg, 2 mL/kg, in physiological saline) without or with C7S (1 or 3 mg/kg). Before and after the treatment, rats consumed feed and water *ad libitum*. Blood samples (approximately 100–150 μL) were collected from the tail vein into heparinized tubes after 2 and 6 h. After 24 h, blood (cardiac puncture) and kidney samples were collected. Plasma and tissue samples were stored at −80 °C until analysis.

In the second experiment, rats were weighed then treated perorally with OTA (100 μg/kg/day, 10 mL/kg, in physiological saline) without or with CHR (1 or 3 mg/kg/day) for three consecutive days. Before and after the treatment, rats consumed feed and water *ad libitum*. In the fourth day, blood (cardiac puncture) and kidney samples were collected. Plasma and tissue samples were stored at −80 °C until analysis.

This study was performed in agreement with the European legislation (Directive 2010/63/EU) and Hungarian Government regulation (40/2013., II. 14.) regarding the protection of animals used for scientific purposes. The experiments were approved by the Ethics Committee on Animal Research of University of Pécs and by the National Scientific Ethical Committee on Animal Experimentation of Hungary and licensed by the Government Office of Baranya County (license No.: BA02/2000–34/2019).

#### 2.5.2 Sample Preparation

Sample preparation was performed as it has been previously reported.^48^ Briefly, after centrifugation, rat plasma was diluted with a 2-fold volume of acetonitrile, and then samples were vortexed, sonicated for 3 min, and centrifuged for 5 min at 14,000*g* and 4 °C. Thereafter, a 2-fold volume of HPLC eluent (see in Section 2.6) was added to the supernatant. These samples were directly analyzed by HPLC-FLD (see Section 2.6).

After weighing, kidney samples were homogenized with a 2-fold amount of water employing a Potter–Elvehjem tissue homogenizer. Then, 100 μL of homogenate was diluted with 2-fold volume of acetonitrile, after which these samples were treated the same way as plasma samples (vortexing, sonication, centrifugation, 2-fold dilution with HPLC eluent, and then HPLC-FLD analysis).

### 2.6 HPLC Analysis

OTA was quantified with the previously reported HPLC-FLD method,^48^ employing an integrated HPLC system (Jasco, Tokyo, Japan): autosampler (AS-4050), binary pump (PU-4180), fluorescence detector (FP-920), and ChromNAV2 software. Briefly, the isocratic elution was performed with 1.0 mL/min flow rate at room temperature, using sodium borate buffer (0.01 M, pH 10.0) and ACN (87:13 v/v%) as the mobile phase. Samples (20 μL) were driven through a SecurityGuard precolumn (C18, 4.0 × 3.0 mm; Phenomenex, Torrance, CA, US) linked to a Kinetex EVO (C18, 150 × 4.6 mm, 5 μm; Phenomenex) analytical column. The fluorescence detection of the mycotoxin was carried out at 383 and 446 nm excitation and emission wavelengths, respectively.

### 2.7 Data Analyses

Data represent means ± the standard error of the mean (SEM) values at least from three independent experiments. Statistical differences were evaluated based on one-way ANOVA and Tukey’s posthoc tests using SPSS Statistics software (IBM, Armonk, NY, US), where the level of significance was set to *p* < 0.05 and *p* < 0.01.

## 3 Results and Discussion

### 3.1 Effects of Site I and Heme Site Ligands on OTA-Albumin Interactions Based on Fluorescence Spectroscopic Studies

First, the fluorescence emission spectra of OTA were recorded in the presence of albumin (HSA or RSA) without and with Site I (warfarin, iodipamide, CHR, or C7S) and Heme site (hemin or bilirubin) ligands. The complex formation of OTA with albumin results in a significant increase in the emission signal of the mycotoxin (λ_ex_ = 393 nm, λ_em_ = 446 nm);^10,12^ therefore, the displacement of OTA from albumin causes a strong decrease in its emission intensity. Under the applied conditions, warfarin, iodipamide, CHR, C7S, hemin, bilirubin, HSA, and RSA did not show any background fluorescence at 446 nm. Before evaluation, the inner-filter effects of warfarin, iodipamide, CHR, C7S, hemin, and bilirubin were corrected (see eq 1) in order to eliminate their absorption-related impacts on the fluorescence signal of OTA.

**Figure 2:**
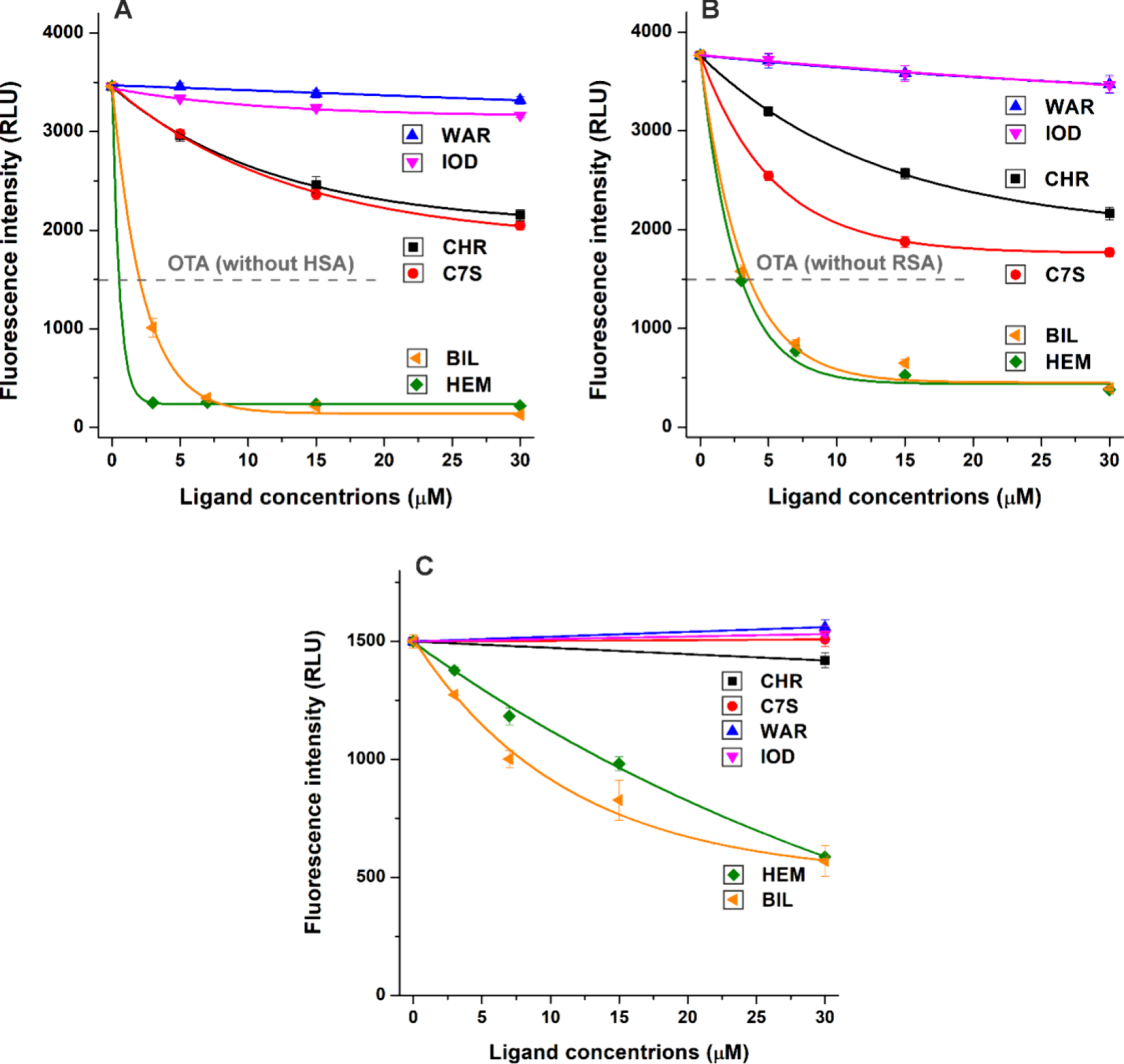
Effects of Site I (warfarin, iodipamide, CHR, and C7S) and Heme site (hemin and bilirubin) ligands (0–30 μM) on the emission signal of OTA (1.0 μM) in the absence and presence of albumin (1.5 μM). Influence of ligand molecules on the fluorescence emission signals of OTA-HSA (A), OTA-RSA (B), and OTA without albumin (C) in PBS (pH 7.4), after correction of their inner-filter effects (λ_ex_ = 393 nm, λ_em_ = 446 nm; *n* = 4; WAR, warfarin; IOD, iodipamide; HEM, hemin; BIL, bilirubin). The representative fluorescence emission spectra of OTA-HSA, OTA-RSA, and OTA samples in the absence and presence of WAR, IOD, CHR, C7S, HEM, or BIL (each 30 μM) are demonstrated in Figure S1.

Warfarin and iodipamide only slightly modified the emission signals of the OTA-HSA (Figure 2A) and OTA-RSA (Figure 2B) complexes. However, CHR and C7S caused strong decreases in the emission intensity of the mycotoxin in the presence of both albumins: the impacts of CHR and C7S were similar regarding the OTA-HSA complex, while C7S caused somewhat stronger reduction in the emission intensity of OTA-RSA samples than CHR. Even at 30 μM concentration, Site I ligands barely affected the emission signal of OTA without albumin (Figure 2C). Therefore, these data suggest that warfarin and iodipamide cannot considerably interfere with OTA-albumin interactions, while CHR and C7S may be able to strongly reduce the albumin-bound fraction of the mycotoxin.

Hemin and bilirubin induced remarkable decreases in the emission signal of OTA in the presence of both albumins (Figure 2A,B). However, these Heme site ligands also strongly reduced the fluorescence intensity of the mycotoxin in the absence of albumin, even after correction of their inner-filter effects (Figure 2C). These observations suggest that the hemin- and bilirubin-induced decreases can be possibly derived from three components: (1) changes in the fluorescence of OTA-albumin complex, (2) changes in the fluorescence of unbound free OTA, (3) and/or changes in the albumin-bound fraction of the mycotoxin. Therefore, we cannot properly evaluate the impacts of hemin and bilirubin on OTA-albumin interactions using these fluorescence intensity-based data.

Fluorescence anisotropy gives information regarding the rotational freedom of fluorophores.^30^ OTA is a small fluorophore molecule with high rotational freedom; thus, the mycotoxin has a low anisotropy value. However, the complex formation of OTA with a large macromolecule (e.g., albumin) leads to the considerable decrease in its rotational freedom, resulting in its strongly increased fluorescence anisotropy.^10,12^ Since HSA and RSA do not exert fluorescence at 446 nm, the albumin-induced strong increase in the anisotropy of OTA is exclusively caused by the interaction of the mycotoxin with the protein.^12^ Considering these principles, the ligand-induced decrease in the anisotropy of OTA suggests the displacement of the mycotoxin from albumin.^13^ Importantly, in the absence of albumin, the fluorescence anisotropy of OTA (*r* = 0.007) was not affected by the Site I and Heme site ligands.

**Figure 3:**
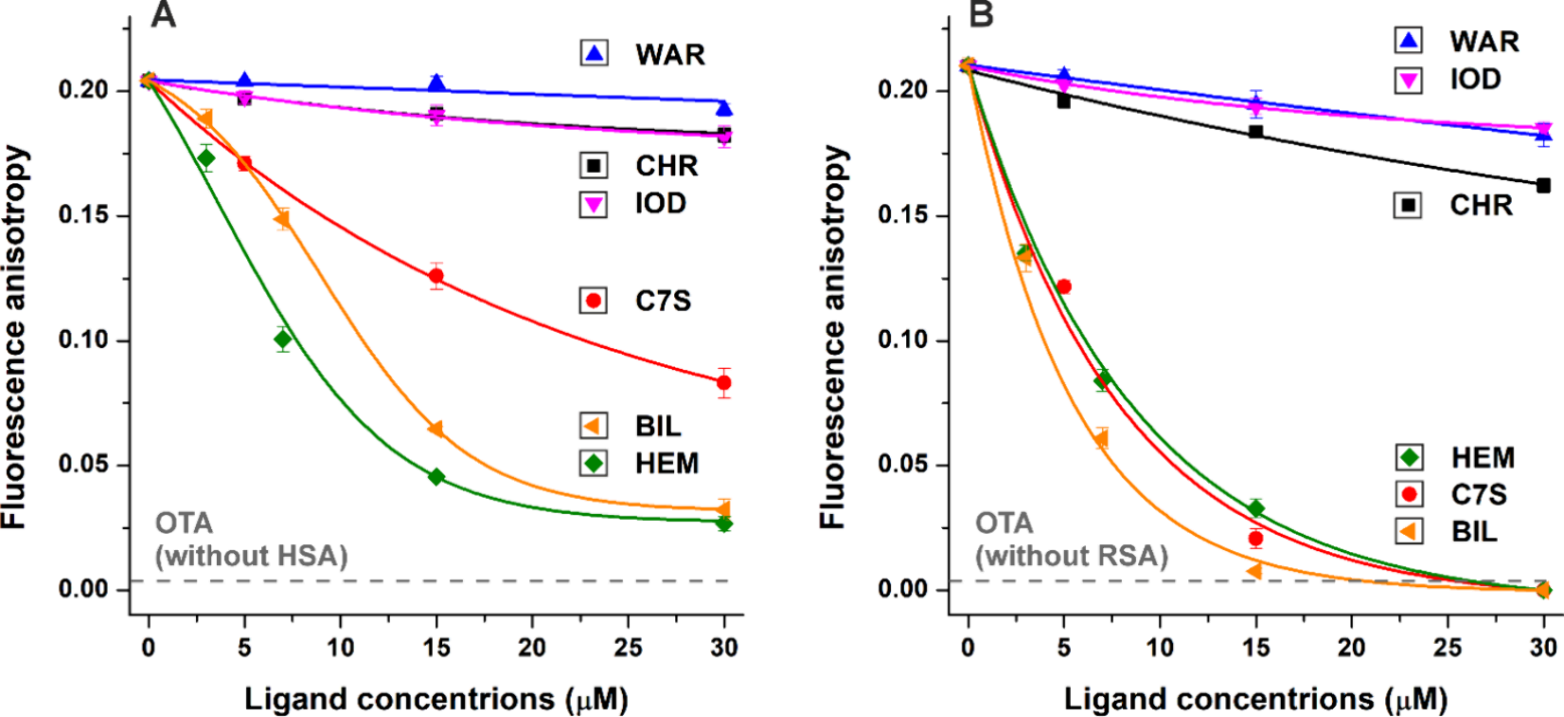
Effects of Site I (warfarin, iodipamide, CHR, and C7S) and Heme site (hemin and bilirubin) ligands (0–30 μM) on the fluorescence anisotropy of OTA (1.0 μM) in the presence of albumin (1.5 μM). Influence of ligand molecules on the fluorescence anisotropy values of OTA-HSA (A) and OTA-RSA (B) complexes in PBS (pH 7.4; λ_ex_ = 393 nm, λ_em_ = 446 nm; *n* = 4; WAR, warfarin; IOD, iodipamide; HEM, hemin; BIL, bilirubin). The fluorescence anisotropy value of OTA (without albumin; *r* = 0.007 ± 0.001) did not change in the presence of Site I and Heme site ligands.

Warfarin, iodipamide, and CHR only slightly decreased the anisotropy of OTA in the presence of HSA or RSA. Interestingly, C7S caused marked reduction in the anisotropy of OTA-HSA (Figure 3A), and it induced an even larger decrease in OTA-RSA samples (Figure 3B). Thus, anisotropy measurements refer to the considerably stronger impact of C7S on OTA-albumin interactions compared to CHR. Considering fluorescence intensity (Figure 2) and anisotropy (Figure 3) data, it is reasonable to hypothesize that CHR can cooperatively bind to the Site I region of albumin with OTA, during which the flavonoid aglycone decreases the emission signal of the OTA-albumin complex without the considerable displacement of the mycotoxin. This assumption is coherent with some previous studies suggested the cooperative binding of certain flavonoid aglycones and warfarin to the Site I region of HSA.^40,49^ Further differences between the results of intensity (Figure 2) and anisotropy (Figure 3) measurements are the smaller, gradual decreases in the anisotropy values in the presence of hemin and bilirubin. Fluorescence anisotropy typically provides more reliable results than intensity-based measurements, referring to the significant displacement of OTA from albumin by C7S, hemin, and bilirubin. On the other hand, anisotropy-based measurements also gave indirect data. Therefore, in the following experiments, ultracentrifugation studies were also performed.

### 3.2 Effects of Site I and Heme Site Ligands on OTA-Albumin Interactions Based on Ultracentrifugation Studies

Using the proper experimental conditions (see details in Section 2.3), albumin can be sedimented by ultracentrifugation without the disruption of ligand–albumin interactions.^26,29,31^ Thereafter, the unbound free concentrations of the ligand molecules can be directly analyzed in the supernatant. First, increasing levels of HSA or RSA (0.0, 0.5, 1.0, and 1.5 μM) were added to the standard concentration of OTA (1.0 μM) in PBS (pH 7.4). After ultracentrifugation, OTA concentrations were quantified in the supernatants by HPLC-FLD (see in Section 2.6). Assuming a 1:1 stoichiometry of complex formation, association constants were determined using eq 3. Based on ultracentrifugation experiments, *K*_*a*_ values of OTA-HSA and OTA-RSA complexes were 2.5 × 10^7^ and 2.9 × 10^6^ L/mol, respectively. These data are in good agreement with the previously reported binding constants of OTA-HSA (1.0 × 10^7^ to 4.0 × 10^7^ L/mol)^10^ and OTA-RSA (1.5 × 10^6^ L/mol)^12^ complexes determined based on fluorescence spectroscopic (quenching, enhancement, and/or anisotropy) studies. In addition, ultracentrifugation experiments also confirmed the earlier finding that HSA binds OTA with much higher affinity compared to RSA,^12^ explaining the considerably longer plasma elimination half-life of OTA in humans vs rats.^14,15^*K*_*a*_ values of OTA-HSA and OTA-RSA complexes were also determined in the presence of 30 μM concentrations of warfarin, iodipamide, CHR, C7S, hemin, or bilirubin (Table S1). These data demonstrate the ligand-induced decreases in the binding affinity of OTA, where we noticed approximately 100-fold lower *K*_*a*_ values of the mycotoxin in the presence of C7S regarding both HSA and RSA.

**Figure 4:**
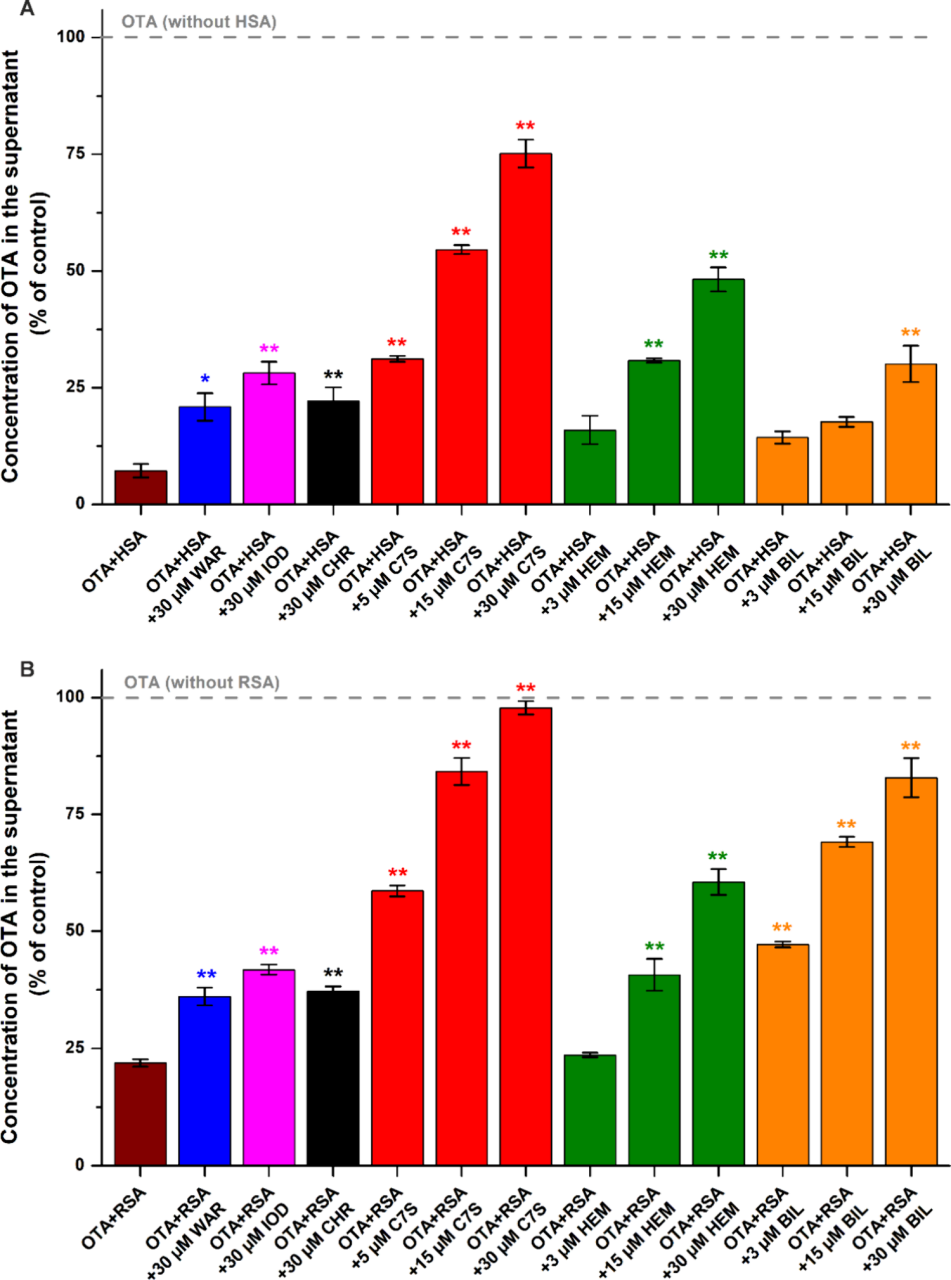
Effects of Site I (warfarin, iodipamide, CHR, and C7S) and Heme site (hemin and bilirubin) ligands on the unbound free fraction (%) of OTA based on ultracentrifugation studies (see details in Section 2.3). Samples contained OTA with HSA (A) or RSA (B) in PBS (pH 7.4), in the absence and presence of the other ligands (OTA: 1.0 μM; albumin: 1.5 μM; site markers: 0–30 μM; *n* = 3; WAR, warfarin; IOD, iodipamide; HEM, hemin; BIL, bilirubin).

In the following experiments, increasing amounts of Site I and Heme site ligands (0–30 μM) were added to standard concentrations of OTA (1.0 μM) and albumin (1.5 μM) in PBS (pH 7.4). Figure 4A demonstrates the concentrations of OTA in the supernatant after ultracentrifugation in the presence of HSA without and with the other ligands tested. The 30 μM concentrations of warfarin, iodipamide, and CHR caused statistically significant but only minor displacement of the mycotoxin from HSA. However, C7S strongly interfered with the OTA-HSA interaction, where the unbound fractions of OTA increased to 55 and 75% in the presence of 15 and 30 μM concentrations of C7S, respectively (Figure 4A). Interestingly, bilirubin caused no significant increase in the free fraction of the mycotoxin at 3 and 15 μM concentrations, and it induced only a minor elevation at 30 μM. Furthermore, hemin showed a better displacing effect than bilirubin, while its impact was considerably weaker compared to C7S (Figure 4A).

Fluorescence intensity (Figure 2A), fluorescence anisotropy (Figure 3A), and ultracentrifugation (Figure 4A) studies were in accordance with warfarin and iodipamide only slightly affect OTA–HSA interaction. The intensity-based experiments with HSA (Figure 2A) suggested that CHR and C7S may have similarly strong displacing abilities, while anisotropy data (Figure 3A) and ultracentrifugation studies (Figure 4A) explored the much stronger displacing effect of C7S.

Fluorescence anisotropy measurements suggested that Heme site ligands may strongly decrease the HSA-bound fraction of OTA (Figure 3A), while ultracentrifugation experiments highlighted the weak to moderate displacement of mycotoxin from HSA in the presence of hemin and bilirubin (Figure 4A). Heme site and Site I are allosterically coupled;^5^ thus, Heme site ligands can modify the 3D structure of the Site I cavity. Interestingly, the allosteric interaction of hemin and bilirubin with OTA considerably reduced the anisotropy values of OTA-HSA samples without the strong displacement of the mycotoxin. For example, at 15 μM, hemin and bilirubin caused marked decreases in fluorescence anisotropy (Figure 3A), while ultracentrifugation studies demonstrated no or only weak displacing effects (Figure 4A). Thus, anisotropy studies showed good predictive value when the ligands occupied the same binding site as OTA (warfarin, iodipamide, CHR, and C7S are also Site I ligands), while we noticed misleading results regarding the Heme site ligands tested (hemin and bilirubin). Therefore, we had to revise and correct our earlier hypothesis that bilirubin can strongly displace OTA from HSA.^13^

Figure 4B represents the concentrations of OTA in the supernatant after ultracentrifugation in the presence of RSA without and with the other ligands examined. Warfarin, iodipamide, and CHR again showed weak displacing effects. However, OTA-RSA interaction was strongly disrupted by C7S, where the 30 μM concentration of the flavonoid metabolite almost completely displaced the mycotoxin from RSA (Figure 4B). Higher levels (15 and 30 μM) of hemin induced weak to moderate impacts. Furthermore, at each concentration tested (3, 15, and 30 μM), bilirubin caused moderate to strong displacement of OTA from RSA, although it was less effective compared with C7S (Figure 4B). Interestingly, the displacing ability of bilirubin showed marked differences when HSA or RSA were applied (Figure 4), which also underline the relevant species-dependent−discrepancies regarding OTA-albumin interactions.^12,14,15^

Considering the above-listed results, C7S can very effectively displace OTA from both HSA and RSA. Even if C7S binds to HSA with 3-fold higher affinity than CHR,^22^ it does not explain the remarkably stronger displacing ability of the sulfate metabolite compared to the parent flavonoid. For a deeper understanding of the differences between OTA-albumin-C7S and OTA-albumin-CHR interactions, molecular modeling studies were performed.

### 3.3 Modeling Studies

Based on the experimental observations collected in the present work and in our earlier study,^22^ molecular modeling was employed to unveil the possible mechanistic rationale underlying the capability of C7S, but not CHR to effectively displace OTA from both HSA and RSA. Another relevant metabolite of CHR is C7G, which also contains a large hydrophilic substituent (similar to C7S); therefore, modeling studies were also carried out with this conjugate.

**Figure 5:**
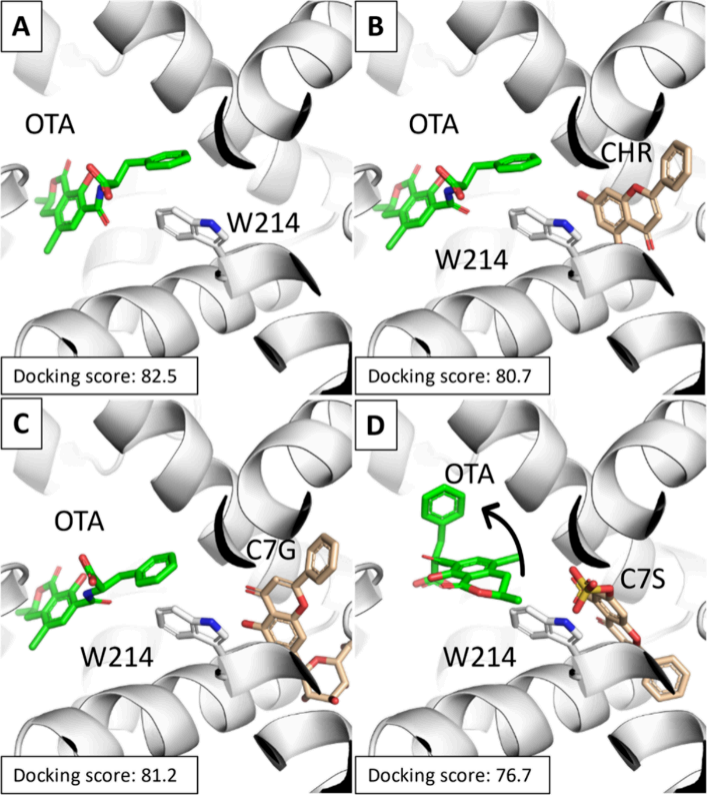
Docking results regarding OTA-HSA (A), OTA-HSA-CHR (B), OTA-HSA-C7G (C), and OTA-HSA-C7S (D) complexes. HSA is represented in white cartoons, while ligands and W214 are represented in sticks.

Once HSA and RSA 3D models were obtained (see Section 2.4.1), they underwent docking studies to provide a reliable architecture of binding for OTA, CHR, C7S, and C7G (Figure 5 and Figure S2). Of note, OTA showed a comparable architecture of binding in HSA and RSA. Subsequently, CHR, C7S, and C7G were docked into HSA (Figure 5) and RSA (Figure S2) in a region of the Site I flanking that occupied by OTA (see in Section 2.4.2).^40^ Importantly, C7S, but not CHR or C7G, caused a diverse arrangement of OTA at Site I, likely due to the steric hindrance of the sulfate moiety (Figure 5D and Figure S2D). Specifically, C7S oriented its sulfate group in both albumins toward the space available to arrange OTA forcing it to adopt a diverse orientation with respect to when it is alone or in complex with CHR or C7G (Figure 5 and Figure S2). Moreover, keeping in mind that docking scores may correlate with the strength of interaction (the lower the score, the worse the fitting), OTA recorded the lowest score in the simultaneous presence of C7S. These data suggest that HSA and RSA are less suitable to bind OTA when also in complex with C7S compared to CHR or C7G.

Afterward, the thermodynamic stability of such complexes was estimated by the HADDOCK Prodigy Web server,^42^ in agreement with our previous study.^43^ The most stable complexes were OTA-HSA and OTA-RSA (−10.3 and −5.9 kcal/mol, respectively), OTA-HSA-CHR and OTA-RSA-CHR (−10.4 and −5.8 kcal/mol, respectively), and OTA-HSA-C7G and OTA-RSA-C7G (−10.5 and −5.8 kcal/mol, respectively), showing comparable Δ*G* values of OTA-albumin complexes in the presence of CHR and C7G. Conversely, in line with the lower docking score recorded, OTA-HSA-C7S and OTA-RSA-C7S showed the highest predicted Δ*G* values (−9.9 and −5.4 kcal/mol), pointing to the less favored interaction of OTA with albumin when C7S is also bound.

**Figure 6:**
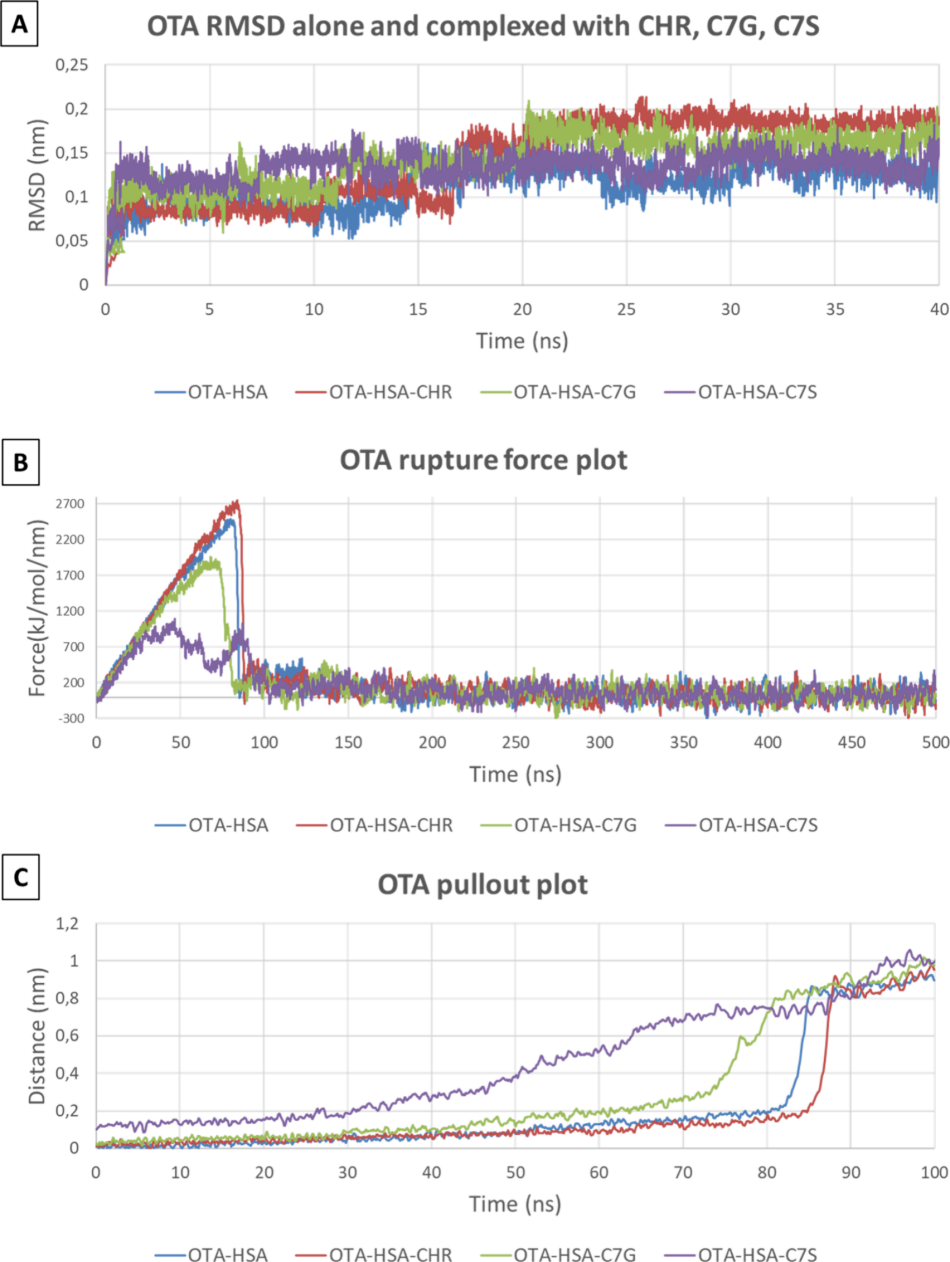
Results of CMD showing the RMSD of OTA when in complex with HSA alone or along with CHR, C7G, or C7S (A). Results of SMD: Rupture force plot showing the lowest energy to detach OTA from the HSA-C7S complex compared to HSA, HSA-CHR, and HSA-C7G (B); and focus on the first 100 ps pullout plot showing the earlier and continuous OTA outward pathway when complexed with HSA-C7S compared to HSA, HSA-CHR, and HSA-C7G (C).

Then, MD simulations were used to provide a detailed mechanistic analysis of HSA being relevant from a real-world food safety perspective. Specifically, CMD simulations were run first. The analysis of OTA trajectories and geometrical stability in the various complexes did not show appreciable differences (Figure 6A) pointing to its overall geometrical stability. This is in line with the actual capability of OTA to bind HSA, although it could be displaced when C7S is also bound. Therefore, the capability of C7S to displace OTA was investigated by means of SMD. Specifically, the rupture force needed to detach OTA from HSA and the related pullout plot were investigated. As Figure 6 demonstrates, OTA-HSA, OTA-HSA-CHR, and OTA-HSA-C7G were almost comparable for both considered parameters, while OTA-HSA-C7S showed a much lower rupture force (Figure 6B) associated with a much smoother pullout plot (Figure 6C and Figure S3). This indicated that CHR and C7G could not appreciably affect the detachment of OTA from HSA, while the altered binding pose caused by C7S could facilitate its outward pathway. These results suggest that the alternative occupancy of Site I due to the presence of C7S made OTA more prone to get displaced. This is in line with the experimentally described capability of C7S to decrease the albumin-bound fraction of OTA (Figure 4) and may provide a mechanistic explanation.

### 3.4 Effect of C7S on the Plasma and Kidney Levels of OTA in Rats after Their Single Intravenous Administration

**Figure 7:**
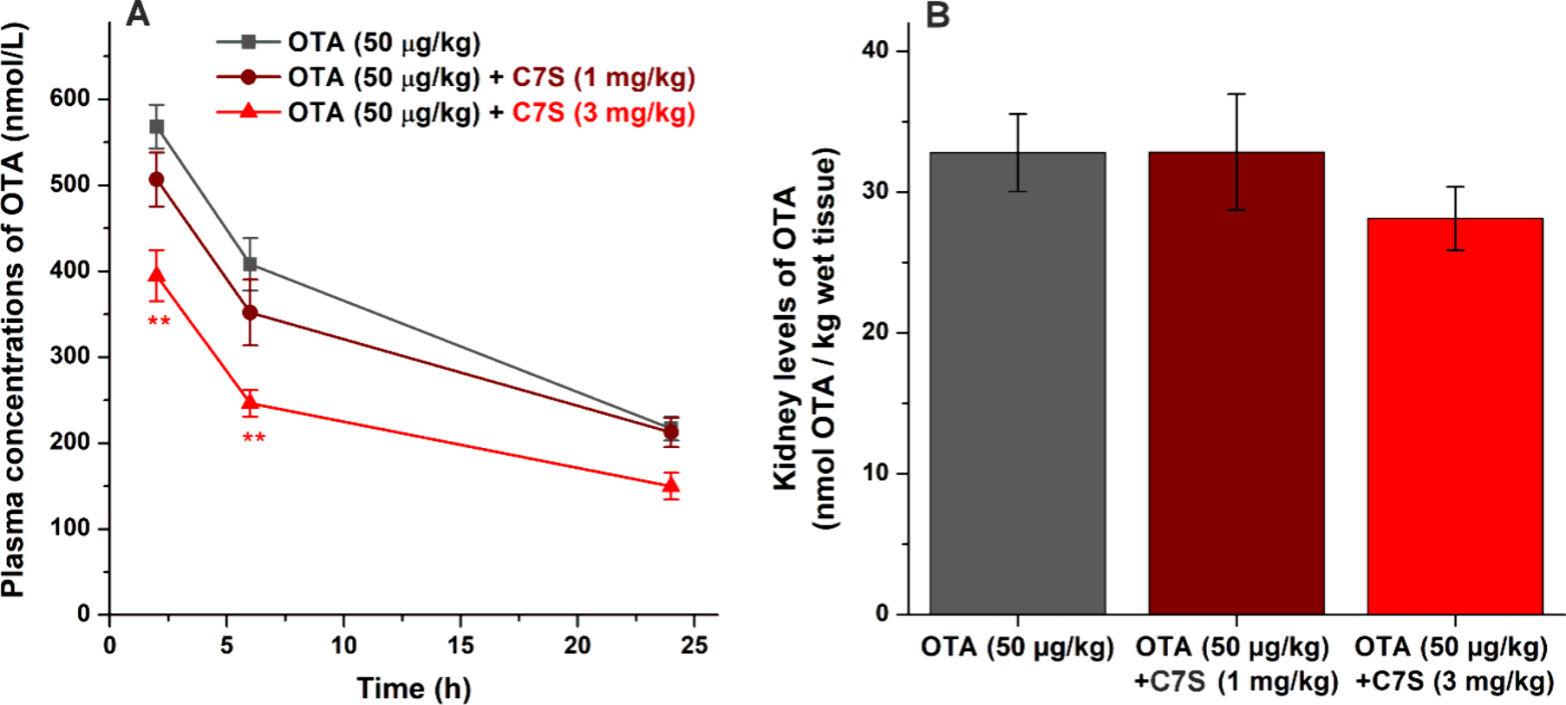
Plasma concentrations (A) and kidney levels (B) of OTA in rats treated intravenously with OTA (50 μg/kg, 2 mL/kg, in physiological saline) alone or cotreated with C7S (1 or 3 mg/kg). Blood samples were taken after 2, 6, and 24 h; kidneys were collected 24 h after the treatment (*n* = 7; ** *p* < 0.01).

In the *i.v.* experiments, we tested the impact of C7S because of the following reasons: (1) CHR is poorly soluble in water, and it is also very difficult to solubilize this flavonoid aglycone at high concentrations; (2) in circulation, C7S is one of the most dominant metabolites of CHR. A single dose of OTA (50 μg/kg) without or with C7S (1 mg/kg or 3 mg/kg) was administered intravenously to Wistar rats. After the treatment, blood (2, 6, and 24 h) and kidney samples (24 h) were collected, and then the concentrations of OTA were quantified in rat plasma and kidneys (Figure 7). The 1 mg/kg dose of C7S did not induce significant changes in the plasma levels of OTA, only slight decreases were noticed after 2 and 6 h, which completely disappeared when the last blood samples were collected (24 h). However, the 3 mg/kg dose of the flavonoid metabolite significantly (*p* < 0.01) decreased the circulating concentration of OTA in the first two time points, while the difference was not statistically significant after 24 h (Figure 7A). These data demonstrate that C7S was able to disrupt the toxicokinetics of OTA, likely due to the displacement of mycotoxin from albumin. Nevertheless, this early impact was countervailed later by certain compensatory effects (e.g., the active reabsorption of the mycotoxin).

The displacement of OTA from HSA can be beneficial if it makes faster the excretion of the mycotoxin from the body, while the increased free fraction of OTA can be harmful if it significantly increases the tissue uptake of the mycotoxin.^17^ Therefore, another important question was the impact of C7S on kidney levels of the mycotoxin. The lower dose (1 mg/kg) of C7S did not affect the concentrations of OTA in kidneys, but its higher dose (3 mg/kg) slightly decreased them (Figure 7B). Even if the difference noticed is not statistically significant, our results suggest that the moderate displacement of OTA from albumin does not cause higher accumulation of mycotoxin in the kidneys.

Importantly, as a limitation of our study, C7S may also interact with other proteins involved in the toxicokinetics of OTA. For example, flavonoids (e.g., CHR) can inhibit OAT1,^50^ which transporter takes part in the active uptake of OTA into kidney cells.^18,19^ Therefore, we cannot be sure that the observed *in vivo* effects can be exclusively attributed to the C7S-mediated displacement of OTA from serum albumin.

### 3.5 Effects of CHR on the Plasma and Kidney Levels of OTA in Rats after Their Repeated Peroral Administration

**Figure 8:**
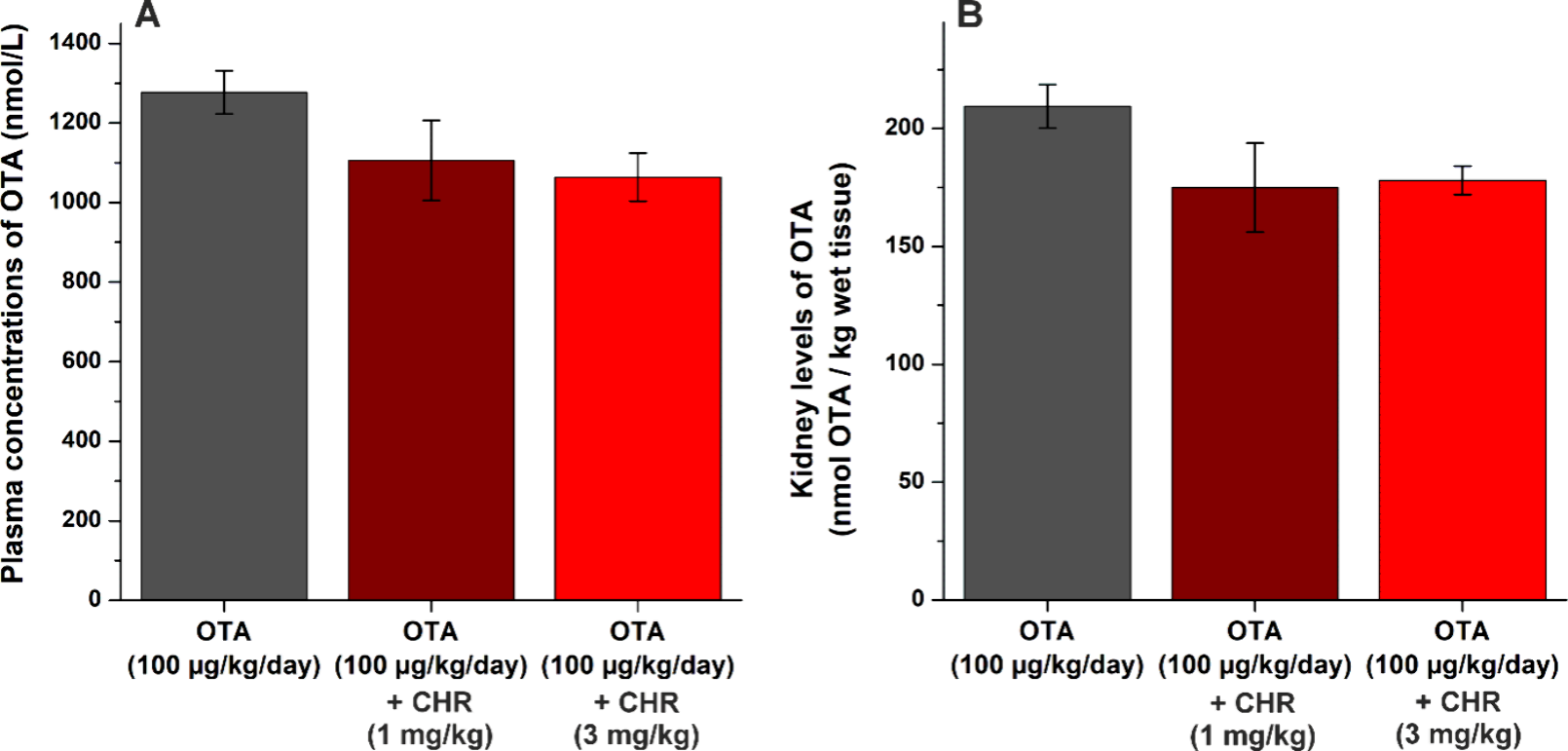
Plasma concentrations (A) and kidney levels (B) of OTA in rats treated perorally with the mycotoxin (100 μg/kg/day, 10 mL/kg, in physiological saline) alone or cotreated with CHR (1 or 3 mg/kg/day) for three consecutive days. In the fourth day, blood samples and kidneys were collected (*n* = 8).

In the *per os* experiments, we examined the effect of CHR because of the following reasons: (1) C7S is more hydrophilic than CHR, therefore, its absorption from the gastrointestinal tract is very uncertain; (2) food and dietary supplements contain CHR, and thus, its *per os* administration is more realistic. OTA (100 μg/kg/day) was administered perorally without or with the parent flavonoid CHR (1 or 3 mg/kg/day) for three consecutive days. At the fourth day, blood and kidney samples were collected and OTA levels were quantified. CHR did not cause statistically significant changes; it only slightly decreased plasma and kidney levels of OTA without clear dose-dependence (Figure 8). These results indicate that even the repeated *per os* coadministration of CHR with OTA does not induce relevant changes in the toxicokinetics of the mycotoxin. Nevertheless, it may be partly resulted from the incomplete absorption of the flavonoid aglycone from the gastrointestinal tract and/or the too low amounts of C7S produced.

## 4 Conclusions

In summary, the displacement of OTA from albumins (HSA and RSA) by Site I and Heme site ligands were examined *in vitro* applying fluorescence spectroscopic, ultracentrifugation, and molecular modeling studies. In addition, the impacts of C7S (*i.v.*, single dose) and CHR (*per os*, repeated administration) on the plasma and kidney levels of OTA were tested in Wistar rats. Fluorescence studies suggested the very strong displacing ability of the Heme site ligands hemin and bilirubin. However, ultracentrifugation experiments highlighted that hemin can moderately decrease the bound fraction of OTA, while bilirubin showed strong impacts only in the OTA-RSA samples. Both fluorescence anisotropy and ultracentrifugation studies demonstrated that C7S can strongly decrease the albumin-bound fraction of the mycotoxin, while the parent flavonoid CHR (as well as the other Site I ligands warfarin and iodipamide) had much lower displacing effects. Based on modeling studies, the simultaneous binding of C7S and OTA to albumin forces an altered binding mode of the mycotoxin with both HSA and RSA. This different arrangement of OTA within HSA or RSA can facilitate the detachment of the mycotoxin as demonstrated by the SMD results and the experimental observations. The repeated *per os* coadministration of CHR for three consecutive days slightly reduced OTA levels but did not induce significant (*p* < 0.05) changes in the concentrations of the mycotoxin in rat plasma and kidneys. However, 2 and 6 h after the *i.v.* treatment with C7S (3 mg/kg) and OTA, we noticed considerably lower plasma levels of the mycotoxin in C7S-cotreated animals (*p* < 0.01). After 24 h, C7S (*i.v.*, 3 mg/kg) cotreatment resulted in somewhat lower concentrations of OTA in plasma and kidneys; although, these differences were not statistically significant (*p* < 0.05). Our data demonstrate the superior displacing effects of C7S vs OTA regarding both HSA and RSA. In addition, C7S decreased plasma levels of OTA without the elevated uptake of the mycotoxin into kidney cells, while later certain compensatory mechanisms reduced the C7S-induced changes. This study shows that we can effectively attack OTA-albumin complexation with certain nonconventional Site I ligands (e.g., C7S); however, even the strong *in vitro* displacing ability does not guarantee highly relevant *in vivo* outcomes. Importantly, the C7S-induced (3 mg/kg) considerable (2–6 h) and minor (after 24 h) changes in the plasma concentrations of OTA did not increase the kidney levels of the mycotoxin. This observation may suggest that the moderate displacement of OTA from albumin does not lead to accumulation of the mycotoxin in the main target organ. However, further studies (with other novel and highly effective competitors) are required to confirm this hypothesis.

## Supporting Information Available

Fluorescence emission spectra of OTA-HSA, OTA-RSA, and OTA samples without and with warfarin, iodipamide, CHR, C7S, hemin, or bilirubin; *K*_a_ values of OTA-HSA and OTA-RSA complexes in the absence and presence of warfarin, iodipamide, CHR, C7S, hemin, or bilirubin; docking results of OTA within RSA, OTA-RSA-CHR, OTA-RSA-C7G, and OTA-RSA-C7S complexes; and the results of SMD complete pullout plots regarding OTA-HSA, OTA-HSA-CHR, OTA-HSA-C7G, and OTA-HSA-C7S complexes (PDF)
