# Supplementary material for: Effects of Chrysin and Chrysin-7-sulfate on Ochratoxin A-Albumin Interactions and on the Plasma and Kidney Levels of the Mycotoxin in Rats
Source: ACS Omega. 2024 Apr 2;9(15):17655–66. doi: 10.1021/acsomega.4c01738 (PMC11024961; doi:10.1021/acsomega.4c01738)
Supplement: Supplementary file 1 — ao4c01738_si_001.pdf [file ao4c01738_si_001.pdf]

Effects of chrysin and chrysin-7-sulfate on ochratoxin A-albumin interactions and on the plasma and kidney levels of the mycotoxin in rats

SUPPLEMENTARY MATERIAL

Miklós Poór [1,2,3*], Ágnes Dombi [3], Eszter Fliszár-Nyúl [3], Lorenzo Pedroni [4], Luca Dellafiora [4]

[1] Department of Laboratory Medicine, Medical School, University of Pécs, Ifjúság útja 13, H-7624 Pécs, Hungary

[2] Molecular Medicine Research Group, János Szentágothai Research Centre, University of Pécs, Ifjúság útja 20, H-7624 Pécs, Hungary

[3] Department of Pharmacology, Faculty of Pharmacy, University of Pécs, Rókus u. 2, H-7624 Pécs, Hungary

[4] Department of Food and Drug, University of Parma, Via G.P. Usberti 27/A, 43124 Parma, Italy

*Corresponding author: Miklós Poór, PharmD, PhD
Department of Laboratory Medicine,
Medical School, University of Pécs
Ifjúság útja 13, H-7624 Pécs, Hungary
Phone: +36-72-501-500 ext: 29250
E-mail: poor.miklos@pte.hu

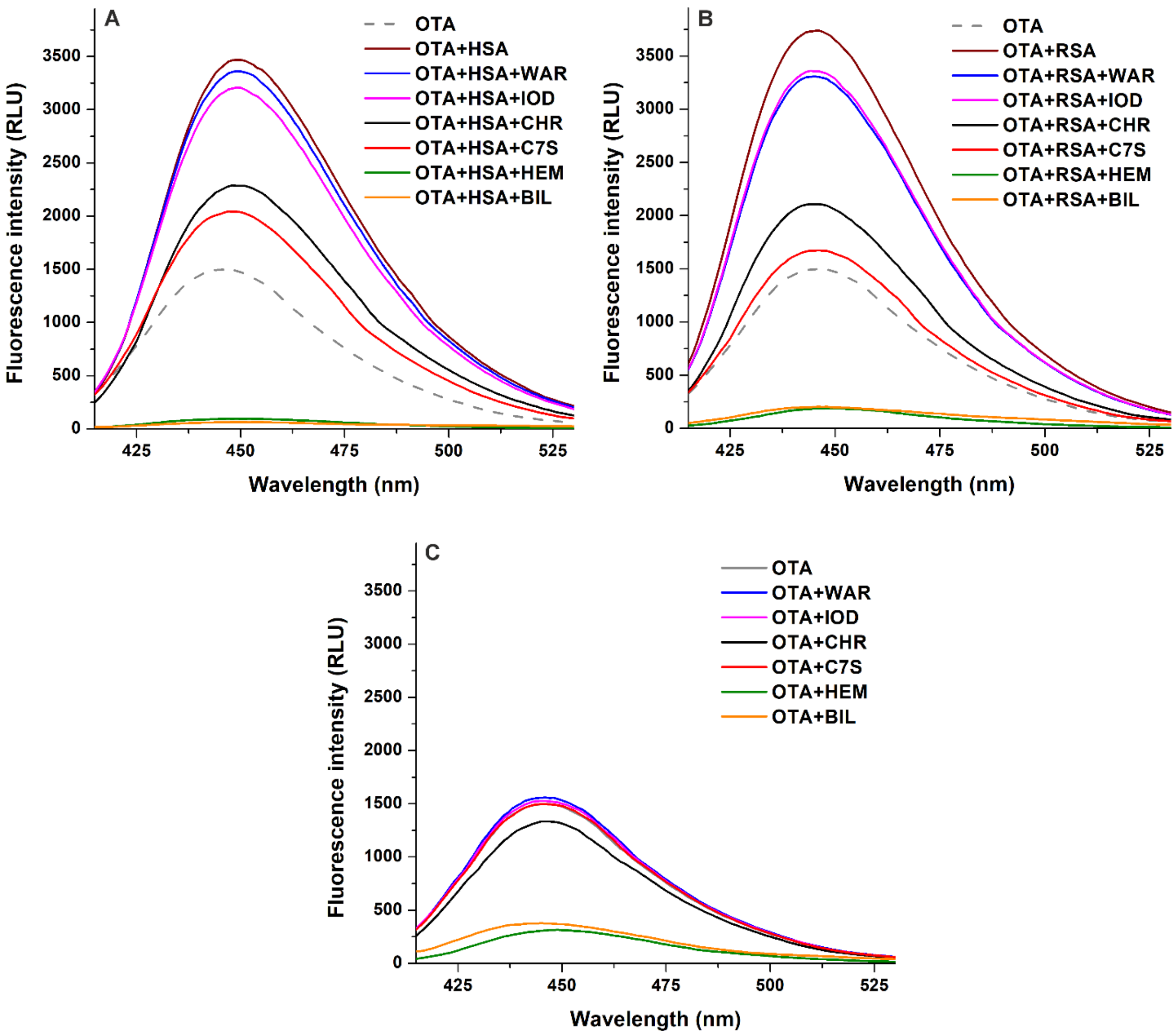

Figure S1: Representative fluorescence emission spectra of OTA-HSA (1.0 μM and 1.5 μM, respectively; **A**), OTA-RSA (1.0 μM and 1.5 μM, respectively; **B**), and OTA (1.0 μM; **C**) samples without and with 30 μM concentrations of warfarin (WAR), iodipamide (IOD), CHR, C7S, hemin (HEM), or bilirubin (BIL) in PBS (pH 7.4; λ_{ex} = 393 nm).

Table S1: K_a values (determined from ultracentrifugation data based on Equation 3) of OTA-HSA (1.0 μM and 1.5 μM, respectively) and OTA-RSA (1.0 μM and 1.5 μM, respectively) complexes in the absence and presence of 30 μM of warfarin (WAR), iodipamide (IOD), CHR, C7S, hemin (HEM), or bilirubin (BIL).

**OTA-HSA**		**OTA-RSA**	
**Sample**	$\boldsymbol{K_a}$	**Sample**	$\boldsymbol{K_a}$
OTA-HSA	2.45×10^7 L/mol	OTA-RSA	2.94×10^6 L/mol
OTA-HSA + WAR	5.35×10^6 L/mol	OTA-RSA + WAR	1.52×10^6 L/mol
OTA-HSA + IOD	3.27×10^6 L/mol	OTA-RSA + IOD	1.10×10^6 L/mol
OTA-HSA + CHR	4.88×10^6 L/mol	OTA-RSA + CHR	1.42×10^6 L/mol
OTA-HSA + C7S	2.64×10^5 L/mol	OTA-RSA + C7S	1.17×10^4 L/mol
OTA-HSA + HEM	1.09×10^6 L/mol	OTA-RSA + HEM	4.55×10^5 L/mol
OTA-HSA + BIL	2.90×10^6 L/mol	OTA-RSA + BIL	1.20×10^5 L/mol

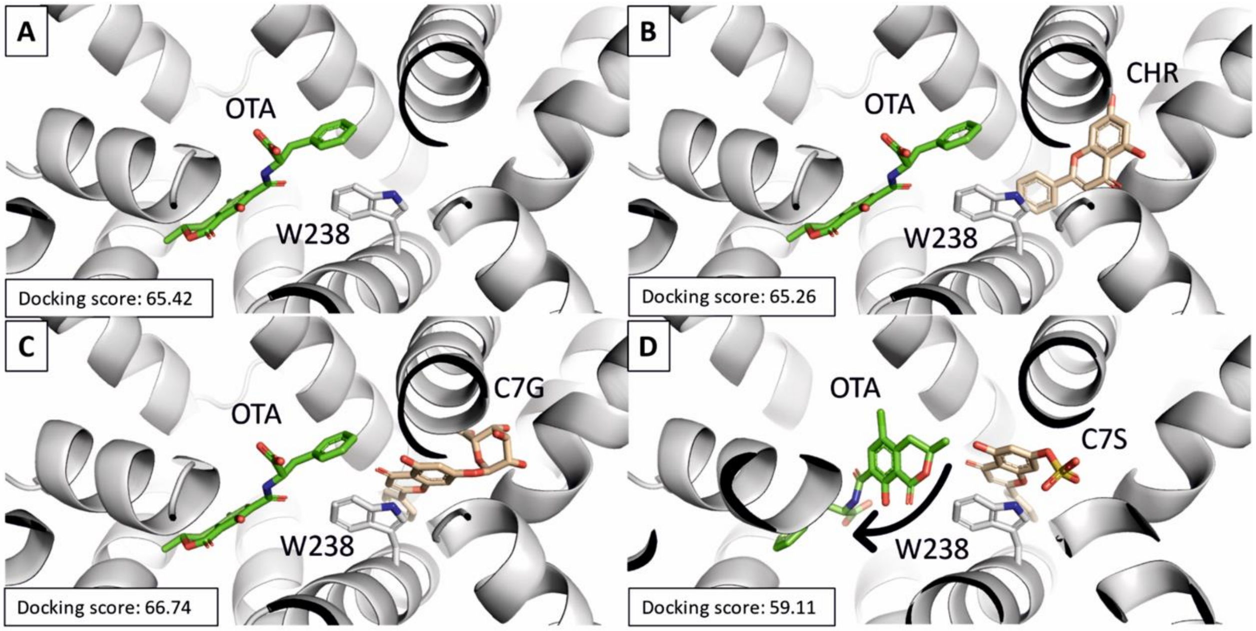

Figure S2: Docking results of OTA within RSA (**A**), OTA-RSA-CHR (**B**), OTA-RSA-C7G (**C**), and OTA-RSA-C7S (**D**) complexes. RSA is represented in white cartoons, while ligands and W238 are in sticks.

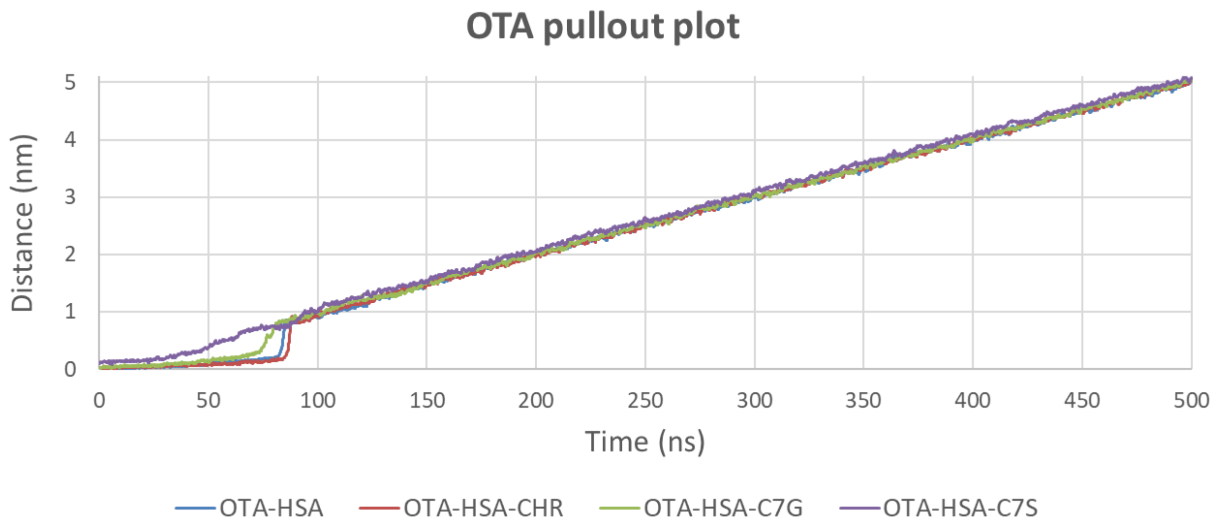

Figure S3: The results of SMD complete pullout plots regarding OTA-HSA, OTA-HSA-CHR, OTA-HSA-C7G, and OTA-HSA-C7S complexes.
